# Leveraging dynamic enhancer landscapes to decode microglia phenotypes in Alzheimer’s disease

**DOI:** 10.1002/alz.090381

**Published:** 2025-01-03

**Authors:** Christopher K. Glass

**Affiliations:** ^1^ University of California, San Diego, San Diego, CA USA

## Abstract

**Background:**

Microglia are the major innate immune cells of the brain and play diverse roles in brain development and homeostasis. In the context of Alzheimer’s disease, microglia acquire new phenotypes that can exert protective or pathogenic roles. Single cell and single nuclei RNA sequencing experiments have defined molecular signatures of different disease‐associated microglia states associated with protective or pathogenic functions, but the mechanisms driving these transitions are not known. As a general approach to this question, we are performing a quantitative analysis of the epigenetic landscapes of mouse and human microglia subsets in models of amyloid pathology.

**Method:**

In collaboration with the Butovsky and Henna laboratories, we defined amyloid responsive enhancers in aged APP/PS1 mice using ATAC seq. In collaboration with the Blurton‐Jones laboratory, we used ATAC‐seq to perform quantitative analysis of enhancers in homeostatic, MHCII‐high and CD9‐high human microglia engrafted into humanized 5XFAD mice. We integrated these findings with patterns of transcription factor expression and changes in gene expression.

**Result:**

The responses of both mouse and human microglia to amyloid pathology in vivo was associated with significant increases in ATAC‐seq signal at more than 1400 genomic locations. A large fraction of these locations correspond to putative enhancers. More than ten distinct transcription factor motifs were identified in both mouse and human amyloid responsive ATAC‐seq peaks. There was a near complete overlap of these motifs in both mouse and human microglia, which were associated with a similar set of expressed transcription factors. A subset of these factors was evaluated for their potential roles in regulating amyloid responsive genes using human iPSC derived microglia and mouse macrophage systems. Although the transcription factor motifs identified in amyloid responsive ATAC‐seq peaks were nearly identical between mouse and human microglia, there was substantial divergence in the downstream genes they regulated.

**Conclusion:**

These studies indicate that the response to amyloid involves many transcription factors, nominates specific factors for further study, and reinforces the value of engrafting human microglia into disease models as a means of investigating the impact of human‐specific non coding regulatory elements.

